# Flextory: Flexible Software Factory of IoT Data Consumers

**DOI:** 10.3390/s24082550

**Published:** 2024-04-16

**Authors:** Rafael López-Gómez, Laura Panizo, María-del-Mar Gallardo

**Affiliations:** ITIS Software, Andalucía Tech, Universidad de Málaga, 29071 Malaga, Spain; laurapanizo@uma.es (L.P.); mdgallardo@uma.es (M.-d.-M.G.)

**Keywords:** Internet of Things, software factory, message broker

## Abstract

The success of the Internet of Things (IoT) has driven the development, among others, of many different software architectures for producing, processing, and analyzing heterogeneous data. In many cases, IoT applications share common features, such as the use of a platform or middleware, also known as *message broker*, that collects and manages data traffic between endpoints. However, in general, data processing is very dependent on the case study (sensors that send temperature data, drones that send images, etc.). Thus, the applications responsible for receiving and processing data, which we call *consumers*, have to be built ad hoc, since some of their elements have to be specially configured to solve specific needs of the case study. This paper presents Flextory, a *software factory tool* to make it easier for IoT developers to *automatically* construct configurable *consumer* applications, which we call *FLEX-consumers*. Flextory guides developers through the process of generating Java *consumers* by selecting some desired features such as, for instance, the particular communication protocol to be used. This way, the developer only has to concentrate on designing the algorithm to process the data. In short, the use of Flextory will result in *consumer* applications with configurable behavior, namely *FLEX-consumers*, that can connect to a messaging server (for example RabbitMQ) and process the received messages.

## 1. Introduction

The Internet of Things (IoT) can be defined as the interconnection of heterogeneous devices through the Internet. With the evolution of wireless networks, in particular the fifth generation of mobile networks (5G), IoT has become an enabling technology for a large set of applications from many different domains, such as smart cities, smart farming, Industry 4.0, and e-Health [1]. In IoT applications, at least three main actors can be identified: the source of data (the *producers*/*publishers*), such as sensors that produce information, the processors (the *consumers*) that analyze and transform data following some criteria, and the *actuators* that respond properly according to the information registered.

Despite the wide variety of IoT architectures, most of them rely on an intermediate platform (middleware), called *message broker*, that abstracts the data transmission between IoT peer devices. Brokers support reliable communication and, additionally, can hide the existence of devices connected at any given time. Thus, the communication endpoints (the *producer* and *consumer* nodes) are only self-aware. Thanks to this feature, it is possible to develop loosely coupled and scalable IoT applications. Traditionally, when a developer faces the task of constructing an IoT application for a given domain following this architecture, he/she has to select the intermediate platform that will connect the source and target entities, implement software modules that carry out this communication, and design the software component that processes the data produced by the data source.

Currently, there is a wide variety of general purpose brokers, such as RabbitMQ, Apache Kafka, and Mosquito. One of the main differences between them is the communication protocols used to connect the broker with the *producer* and *consumer* applications. In the IoT domain, brokers usually make use of the two well-known communication protocols AMQP [2] and MQTT [3] that take into account the limited resources of many IoT devices. It is worth mentioning that some broker systems support both communication protocols.

In this work, we have followed the ideas of *Software Product Line Engineering* (SPL) [4], a research area whose aim is to model families of software products that can be used to generate software adapted to the final user’s needs. The SPL community has addressed multiple problems of the IoT domain, such as the design of App Stores that support the deployment of customized applications [5] and the self-adaptation of applications running in heterogeneous IoT devices [6].

The term *software factory* is not new [7] and refers to the set of techniques and tools that automate and simplify software creation [8]. They have been traditionally used to develop industrial applications. Nowadays, they are part of the techniques of SPL Engineering and are broadly used to customize software products of different domains [9]. In particular, we have constructed the tool Flextory that allows the development of IoT *consumer* applications with several facilities. The tool is able to *automatically* construct the software components to read the data from the producer. In addition, Flextory allows the easy integration of any data processing algorithm in the IoT application. This characteristic is very interesting, since it means that the developer only has to focus on the design of the algorithm suitable for the application being implemented. The tasks related to the integration of the algorithm with the rest of the application’s software modules are already provided by the tool. To the best of our knowledge, in the literature, there are no tools similar to Flextory that generate *consumer* applications with flexible behavior in the IoT domain.

The objective of this paper is to describe Flextory in detail. Flextory generates *consumer* applications able to read and process data received from a message broker system. To avoid confusion due to names, we use the term *FLEX-consumer* for the *consumer* applications built with Flextory. As commented above, *FLEX-consumers* have a flexible behavior with many configurable options to ease their deployment. All *FLEX-consumers* have been developed in Java, which is well known for its portability. In addition, Java is also supported by some devices with low computing power [10].

**Figure 1:**
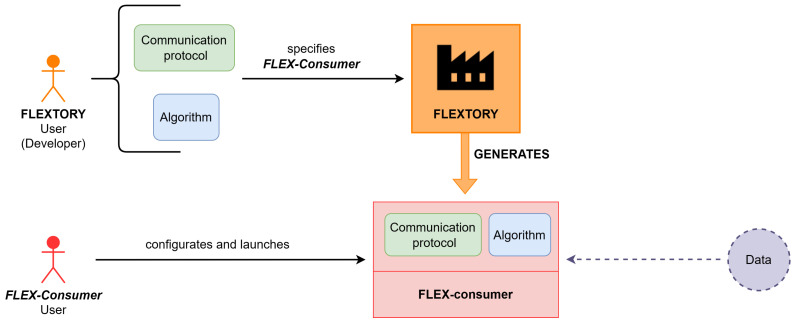
Overview of Flextory and *FLEX-consumer* proposal.

Figure 1 depicts the interaction between the tool Flextory and *FLEX-consumers* and their users. As shown, two different users appear in the diagram. On the one hand, the Flextory user (called developer) makes use of Flextory to construct *FLEX-consumers*. The developer introduces some parameters needed to construct the desired *FLEX-consumer*, such as, for instance, the communication protocol to be used and the algorithm that will process data. On the other hand, the *FLEX-consumer* user configures the application in order to adapt its execution to the particular expected behavior. Usually, the *FLEX-consumer* configuration is related to how and when the data processing algorithm must be executed.

To demonstrate the utility of Flextory and *FLEX-consumers* in the development and deployment of IoT *consumer* applications, we present a non-trivial case study related to the learning technique of black-box systems [11]. Flextory is used to produce a *FLEX-consumer* that reads a sequence of observations and runs a learning algorithm that is able to produce models of the system. In particular, in the case study, the *FLEX-consumer* is used to learn models of the DASH protocols [12]. This example shows how Flextory facilitates the construction of complex IoT applications. As commented above, the developer only has to focus on the construction of Java classes that carry out the learning process. Finally, we have evaluated the tool with a user study in which a group of post-graduate students have performed a task with Flextory and have filled out a questionnaire.

The tool and the documentation is publicly available at https://gitlab.com/morse-uma/formal-methods/flextory/, accessed on 28 March 2024.

The rest of the paper is organized as follows. First, Section 2 introduces some of the most common IoT communication protocols and message brokers. Then, Section 3 summarizes related work. In Section 4, we describe in detail the design and implementation of Flextory and the generated *FLEX-consumers*. Section 5 and Section 6 present two case studies and the user study results, respectively. Finally, in Section 7 and Section 8, we discuss the strengths and weaknesses of our proposal and summarize conclusions and future work. Additionally, we provide supplementary material in Appendix A, expanding on Section 4 and Section 5 to provide more information on the development and application of Flextory.

## 2. Background

In this section, we first present and compare some of the most widely used IoT communication protocols. Then, we introduce the IoT architecture based on message brokers, which is the basis of the design of the tool Flextory. We recommend that the reader consult a state-of-the-art survey on IoT, such as [1], to gain a broader view of this technology.

### 2.1. IoT Communication Protocols

In general, communication protocols establish a set of rules so that different devices can communicate, as well as the format of the messages exchanged. In the case of the IoT domain, communication protocols have to deal with some specific features. Firstly, they must take into account the large number of devices that can be connected, the different tasks that they may perform, and the disparity in devices’ computation capability. In addition, the IoT architecture should be scalable and flexible; that is, adding or removing devices should not produce noticeable changes in the IoT solution. This requirement entails adopting low coupling between devices. Finally, the security of communications must also be ensured.

**Figure 2:**
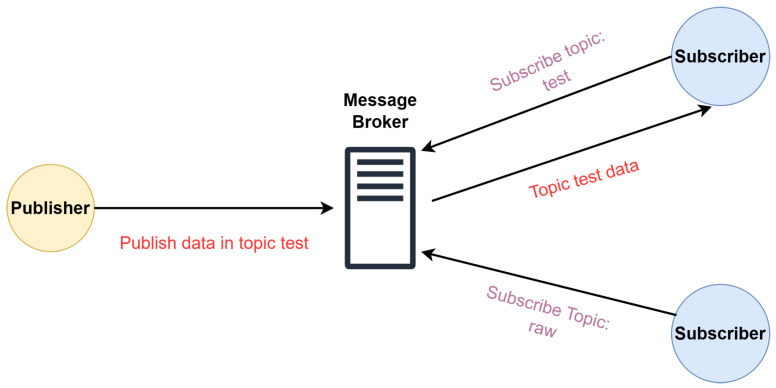
Example of Publish/Subscribe communication pattern.

IoT protocols may be based on different communication patterns, Publish/Subscribe being one of the most widely used. In this communication pattern, there are two entities that interchange data via a middleware broker. On the one hand, the *subscriber* tells a broker the topic of the messages that it wants to receive. On the other hand, the *publisher* sends data about a certain topic to the broker. As shown in Figure 2, the message broker is in charge of distributing the messages to the *subscribers* subscribed to each topic.

We will now describe some IoT protocols based on the Publish/Subscribe pattern that work over the application layer of the OSI network model. Since AMQP and MQTT are, by far, the most widely used protocols, we have decided that developers can configure Flextory to allow the resulting *FLEX-consumer* to make use of one of these two protocols.

- MQ Telemetry Transport [13,14] (MQTT) is an OASIS open standard that defines a Machine to Machine (M2M) communication protocol for IoT environments. MQTT typically works over TCP and transports binary data. MQTT is designed to be lightweight so that it can be used by devices with low computing capacity. In addition, message headers are small to accommodate low bandwidth networks. Finally, regarding security, different security mechanisms are available, such as encrypting connections using SSL/TLS and authentication.
- Advanced Message Queuing Protocol [15] (AMQP) is also an OASIS open standard. AMQP is designed to support a wide variety of communication patterns. Messages are distributed between the endpoint devices by means of a flexible and complex mechanism based on *exchanges*, which are abstract entities declared by users to which messages are sent. *Exchanges* take a message and route it into queues. Users have to create queues if they do not exist and bind them to a specific *exchange*. The routing algorithm that distributes messages from *exchanges* to queues depends on the *exchange* type and the binding rules. There are many *exchange* types, but in this project, we have used the default *exchange* that is pre-declared by the broker. This *exchange* offers a Publish/Subscribe pattern with one special property that makes it very useful for applications: every queue that is created is automatically bound to the *exchange* with a “topic name” (routing key in AMQP nomenclature) that is the same as the queue name. For example, if a queue with the name “test” is declared, the message broker will bind it to the default exchange using “test” as the topic. AMQP provides authentication and encryption based on SASL and TLS.
- Constrained Application Protocol [16] (CoAP) is a protocol for devices with limited resources that provides a REST model between application endpoints with messages in binary format. Targeting efficiency, CoAP runs over the UDP transport layer protocol to distribute messages in an asynchronous manner. Regarding security, it supports DTLS.
- Extensible Messaging and Presence Protocol [17] (XMPP) is an IETF open standard that uses the Extensible Markup Language (XML) as the data format. XMPP was initially designed for instant messaging services but was later extended to cover different communication patterns, such as Request/Response, Asynchronous Messaging, Publish/Subscribe, event subscription (Observe) and delayed delivery. In terms of security, XMPP supports SASL and TLS. However, the support of end-to-end encryption is a work in progress.

### 2.2. Message Brokers

**Figure 3:**
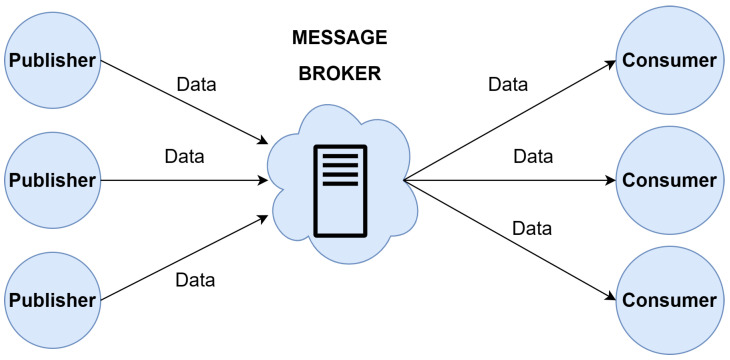
Message broker architecture.

A message broker, or a messaging server, is a middleware between applications or devices, both senders and receivers, that exchange messages. In the IoT context, a message broker consists of a centralized notification service that is a main server with a fixed IP address known by all devices. As shown in Figure 3, the server is responsible for receiving messages from all sending devices (*publishers*) and distributing them to receivers (*consumers*). The connected devices are only aware of themselves. *Consumers* do not know the true origin of the data, and publishers do not know by who or how the data will be processed. This isolation is achieved thanks to the message broker management and provides scalability and low coupling.

Nowadays, a wide variety of message brokers are available, both self-hosted and cloud-hosted. In particular, we have tested the *FLEX-consumers* using RabbitMQ, since it is one of the message brokers that supports MQTT and AMQP. Table 1 shows a comparison between some of the most popular message brokers (despite the fact that all solutions can be deployed in a cloud service provider, we have only considered native cloud hosting). In addition to the type of business model followed, either proprietary or open source, some other differences can be highlighted. For example, the RabbitMQ model has commercial features to gain access to a virtualized version of the message broker and even cloud hosting and technical assistance. Regarding the protocols supported, the brokers accept AMQP and MQTT natively or via plugin, with the exception of Apache Kafka that uses a custom protocol. Finally, Microsoft’s and Amazon’s options also cover the integration and communication with other services provided by these companies.

## 3. Related Work

In the IoT domain, it is common to use the three-layered architecture that distinguishes between the roles of *producers*, message brokers, and *consumers*. One of the main challenges to be addressed when using this architecture is how to adapt it to changes in the data format and the processing algorithm. There exist many proposals that integrate all the components of this three-layered architecture into a custom solution. For example, JCL [23] is a middleware whose purpose is to integrate IoT with High Performance Computing (HPC). It also has an API in which different categories of devices can be programmed. Since JCL requires that its own components be installed in all the systems belonging to the IoT ecosystem, its use is limited to devices that are JCL compatible. Our proposal is focused on end devices that process data (*consumers*) and relies on the portability paradigm of the Java language. In contrast to JCL, *FLEX-consumers* are automatically generated.

D-LITe [24] is another all-in-one solution that uses a choreography approach. Programming is based on cooperation between nodes, each one performing a small part of the total application. To program a node, D-LITe uses finite state machines with an output alphabet, called Finite State Transducers (FSTs), to describe the logic of the application. When a user wants to describe the application, he/she does it using a specific format called SALT. Subsequently, the rules are transformed into a set of FSTs (one per node) that are sent through the network. Finally, each node’s rule analyzer is responsible for executing its FST. The use of D-LITe is limited both by the compatible devices and by programming options offered by the SALT format. As mentioned before, our proposal offers *consumers* with flexible behavior that can be changed in every execution. In addition, there are no programming restrictions because they are built using Java.

IoTSuite [25] also offers a tool suite that covers all the layers that constitute an IoT infrastructure. These tools automatize tasks in different phases of developing an IoT application. For example, programmers can write high-level textual specifications that can be analyzed and transformed into code by the compiler tool. There is also an execution system that incorporates a middleware to coordinate nodes. IoTSuite requires that it be compiled and installed on all devices that will be used, which limits its application.

The platform SYNAISTHISI [26] is another approach to support multiple communication protocols, such as MQTT and AMQP, by integrating different open-source frameworks including, among others, RabbitMQ. This work does not focus on how to support the development of IoT applications. In contrast, its objective is to support the interoperability of these different frameworks and provide a unified user-access control on IoT data and services. The platform is available as a set of dockerized containers; thus, it is easily deployable. Both tools, the SYNAISTHISI framework and Flextory, aim to support the fragmented IoT ecosystem from different approaches. Clearly, the SYNAISTHISI platform can help us to test *FLEX-consumers* generated by Flextory in different scenarios (protocols and brokers) and, alternatively, Flextory can easily produce *FLEX-consumers* with different communication protocols to test the interoperability of SYNAISTHISI.

There are also proposals that only focus on endpoint devices, either *publishers* or *consumers*. For instance, FRASAD [27] is a framework that facilitates the development of programs for sensor nodes (devices physically connected to sensors). FRASAD has been built following a software architecture centered on nodes and a programming model based on rules that allows applications to be described using a language specific to the sensor domain. The application code is generated from models built with the language through an automatic transformation process. FRASAD focuses on the *publisher* part, while our proposal centers on the *consumer* applications.

On the *consumers* side, Midas [28] is a framework to help researchers create and manage IoT applications with heterogeneous data sources. Midas has a module to process the data features of interest by means of the so-called analysis functions that make use of machine learning techniques. The main characteristic of Midas is its modularity, making it easy to incorporate new components in order to add new data streams or analysis functions. In addition, it is implemented as a distributed architecture to assure scalability. Compared with Midas, Flextory’s goal is different, since it is conceived as a meta-tool to create new configurable tools with respect to the structure of input data, the type of message brokers to be used, and the algorithms to process data, among other features.

## 4. Software Description

**Figure 4:**
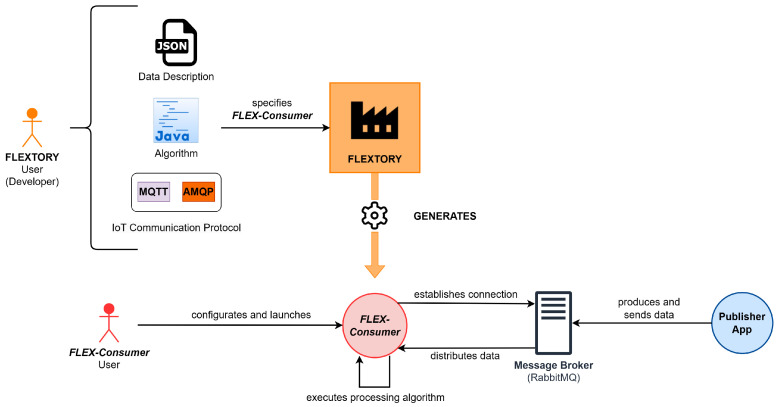
General overview of the inputs and outputs of Flextory and *FLEX-consumers*.

Figure 4 presents a general overview of Flextory and *FLEX-consumers* developed in this work. The top part of the figure shows Flextory’s input and output. Thus, the user of Flextory (a developer of IoT *consumer* applications) introduces parameters that Flextory needs to build a *FLEX-consumer*. The mandatory inputs are the type and structure of the data (in JSON format) that the *FLEX-consumer* will receive from the message broker, the algorithm for data processing, and the communication protocol (AMQP or MQTT) to be used to connect with the message broker. With this information, Flextory automatically generates a *FLEX-consumer*.

The lower part of Figure 4 shows how the resulting *FLEX-consumer* can be configured and executed. Among other functionalities, the *FLEX-consumer* can be configured by the user to connect to a specific message broker and to subscribe to different types of data topics (as described in Section 2.1). Furthermore, there are some other customizable parameters used to establish some execution conditions of the processing algorithm and to decide when the *FLEX-consumer* should stop its execution. We have tested the *FLEX-consumers* generated with Flextory using RabbitMQ as a message broker, but *FLEX-consumers* can connect to any other messaging server supporting AMQP or MQTT protocols.

The rest of the section describes the design of Flextory and the *FLEX-consumers* shown in Figure 4. We first present the design of Flextory, including its main functional and non-functional requirements and some implementation details. Then, we introduce the *FLEX-consumer* requirements related to their configuration.

### 4.1. Design and Implementation of Flextory

As commented before, Flextory automatically creates *consumer* applications with configurable behavior (the *FLEX-consumers*). On the one hand, Flextory must be able to generate *FLEX-consumers* adapted to different IoT domains. This implies that Flextory must allow both different formats for the data received by *FLEX-consumers* and also different processing algorithms to be applied to data. On the other hand, Flextory must construct *FLEX-consumers* able to change the message broker as well as the conditions to trigger the processing algorithm in each execution.

Table 2 contains the list of functional and non-functional requirements that have guided the construction of Flextory. These requirements have been selected to offer developers maximum flexibility when choosing how to build a *FLEX-consumer*. The most relevant requirements are FR-1 to FR-5. FR-1 establishes the need to provide the format of the data received by the *FLEX-consumer*. FR-2 to FR-4 describe the requirements related to the processing algorithm used by the *consumer* applications produced by Flextory. FR-5 is related to the communication protocols to be included in the *FLEX-consumers*.

**Figure 5:**
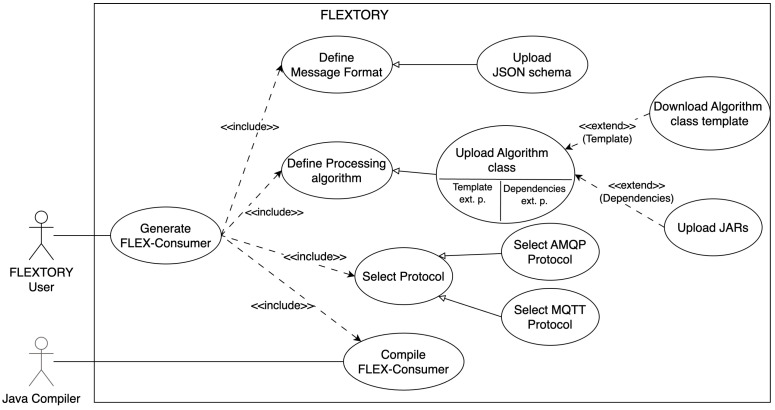
Flextory use case diagram.

Figure 5 shows the use case diagram that describes Flextory’s main capabilities. The main actor is the Flextory user interacting with Flextory to generate a *FLEX-consumer*. To this end, the user uploads the format of the messages to be processed, currently using a JSON schema. In addition, the user defines the processing algorithm. This requires uploading, at a minimum, the implementation of the *Algorithm* class. To ease the process, the user can download a template to be completed. Optionally, if needed, the user can upload external dependencies packaged in JAR format. Moreover, the user must select the communication protocol between the two that are currently available (AMQP and MQTT). Finally, Flextory uses the Java compiler to generate the *FLEX-consumer*, so it is essential that it be installed.

**Figure 6:**
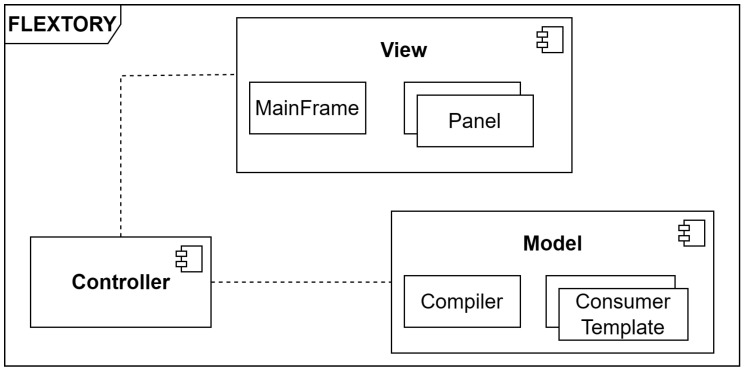
Flextory components diagram implementing Model/View/Controller design pattern.

Figure 6 shows Flextory’s architecture. In Appendix A, we have included supplementary material, such as the class diagram. Flextory follows the classical *Model/View/Controller* design pattern. The *View* module includes the visual components used by the Graphical User Interface (GUI) to guide the user through the configuration and generation of the *FLEX-consumer* application. There are two main visual components: the MainFrame, which provides the skeleton of the Flextory GUI, along with different panels that remain visible during the creation of the *FLEX-consumer* in order to ease the interaction with the user. The *Model* module is in charge of generating the *FLEX-consumer* and is composed of two sub-components, the *Consumer Templates* and the *Compiler*. The former contains the code templates of different *consumer* components, such as the *User Interaction* module or different versions of the *Connection Management* module. The *Compiler* is in charge of integrating the templates with the data provided by the user in order to generate the *FLEX-consumer* executable. Finally, as usual, the *Controller* is the link between the *View* and the *Model*, reacting to user inputs and performing interactions on the *Model*. In addition, it can also react to *Compiler* events to properly update the *View*.

**Figure 7:**
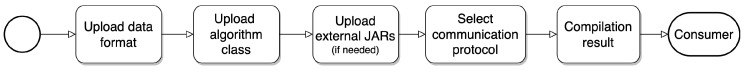
Simplified activity diagram of Flextory.

The current version of Flextory is a Java application with a Java Swing GUI, packed in an executable JAR file. To generate *FLEX-consumers*, Flextory guides users through a sequence of steps shown in the flow diagram in Figure 7. Although it is not explicit in the diagram, the user cannot advance to the next step if the selection made in the current step is wrong. We will use this diagram to present Flextory’s main implementation decisions.

In the first step, the user has to provide a JSON schema describing the format of the data distributed by the message broker. Then, the user uploads the processing algorithm to be used by the *FLEX-consumer*. The algorithm has to be coded in a special Java class called *Algorithm* that implements the Java *Runnable* interface, so that the user only has to implement the *run* method. To make this step easier, Flextory provides a downloadable template of the *Algorithm* class. In addition, if the *Algorithm* class has some dependencies, they have to be provided as JAR files. In the fourth step, the user selects the communication protocol supported by the broker (AMQP or MQTT). With all the necessary files, the *Compiler* module transforms the data description (JSON schema) into a set of Plain Old Java Objects (POJOs) classes that will be part of the *FLEX-consumer*, and will support the deserialization of the data received from the message broker. Finally, the *FLEX-consumer* is built as a Java application that integrates the POJOs classes and the *Algorithm* class with its dependencies and the templates.

To illustrate the use of Flextory, we show how to build a simple application that receives data from the well-known Iris dataset [29] and counts the number of flowers of each species. The dataset has samples of different iris species. Four traits (the length and width of the sepal and petal) are associated with each species. The Flextory user (the developer) has to provide the file with this JSON schema in the first step (see the specific format in Appendix A). Next, the user has to complete the *Algorithm* class to count the number of samples of each species, as show in Listing 1. This algorithm has no external dependencies, so the user, after providing this class, can jump directly to the fourth step and select the communication protocol to generate the *FLEX-consumer* binaries (this example and the generated *FLEX-consumers* are available in gitlab).

**Listing 1.** Implementation of the Algorithm class of the Iris dataset example.

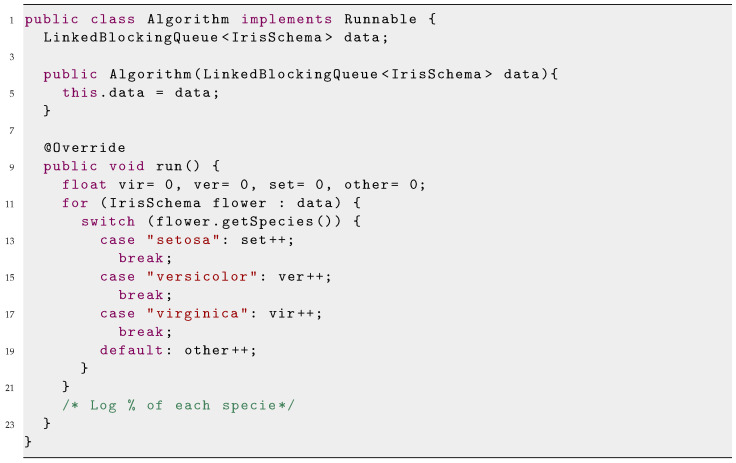



### 4.2. Design and Implementation of FLEX-Consumers

The main purpose of a *consumer* application is to connect to a message broker and process the messages received by applying a processing algorithm. The design of the *FLEX-consumers* takes into account the following aspects. On the one hand, *FLEX-consumers* have to use a standard protocol to communicate with a message broker, such as the AMQP and MQTT protocols introduced in Section 2. To simplify the design, we assume that a *FLEX-consumer* uses only one of these protocols. On the other hand, *FLEX-consumers* can be configured in a *persistent* or *repetition* mode, since the internal behavior of the algorithm is unknown, i.e., it can be designed to process all incoming data in a persistent manner or, on the contrary, to process data batch. In particular, given a processing algorithm, we could define different repetition conditions that establish when the algorithm has to iterate again: each time a new message arrives, when a fixed number of messages are received, or after a specific time has elapsed. In consequence, the design of *FLEX-consumers* allows the configuration of different execution modes for the same algorithm. Moreover, *FLEX-consumers* can be configured with different *halting conditions* that define when the *FLEX-consumer* must close connections and stop the execution. For instance, data processing could stop when a *FLEX-consumer* reaches a maximum number of messages received or when a given time without receiving messages has elapsed.

Considering the foregoing, we have identified the main functional (FR) and non-functional (NFR) requirements of *FLEX-consumers*, which are listed in Table 3. We now describe the most relevant ones. FR-1 to FR-4 define the necessary parameters to establish a connection with a message broker, such as the communication protocol and the topic to subscribe. FR-5 to FR-7 focus on the behavior of the processing algorithm. For example, there will be repetition conditions due to elapsed time or number of messages. FR-8 mentions the need to include options to define conditions of when a *FLEX-consumer* should close connections and end.

**Figure 8:**
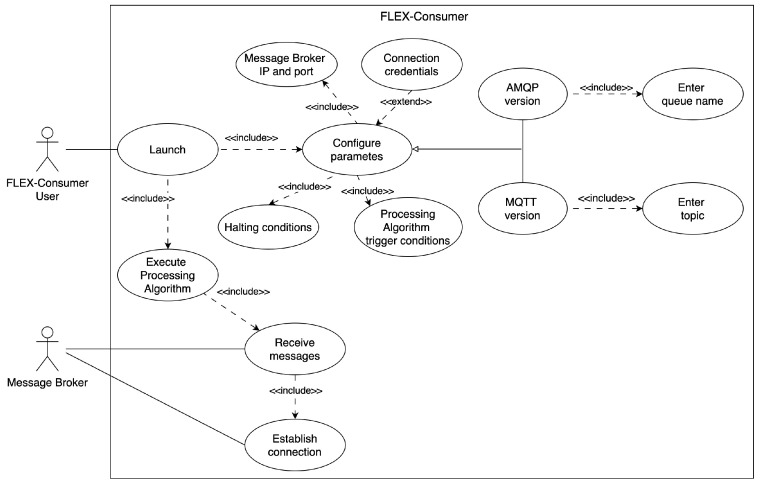
*FLEX-consumer* use case diagram.

Figure 8 shows the use case diagram of a *FLEX-consumer*. The main actor is the *FLEX-consumer* user that launches the consumer in order to process data coming from the message broker. To this end, the user has to configure some mandatory parameters, such as the message broker IP address as well as the queue or topic depending on whether the *FLEX-consumer* uses AMQP or MQTT, and the trigger and halting conditions of the processing algorithm. Additionally, the user can configure connection credentials.

**Figure 9:**
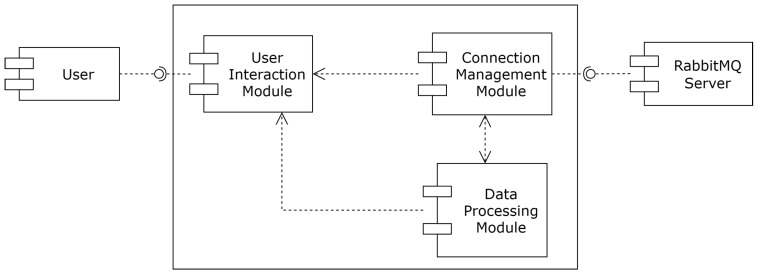
*FLEX-consumer* components diagram.

These requirements lead us to the *FLEX-consumer* architecture shown in Figure 9. A *FLEX-consumer* comprises three main components. The *“User Interaction”* module is responsible for interacting with the user through the command line terminal, mainly to read the configuration parameters and display the results of the processing algorithm, including the errors, if they occur. The *“Connection Management”* module is in charge of establishing and managing the communication with the message broker. Finally, the *“Data Processing”* module deals with the execution of the processing algorithm following the entered configuration. Since this algorithm, which is specific to each *FLEX-consumer*, can have different internal sub-modules, this module can be conceived as a wrapper that controls the algorithm’s execution and stop conditions.

Regarding implementation, *FLEX-consumers* are Java applications in JAR format that are invoked by users using a command line. When *FLEX-consumers* are executed, they receive arguments that define how they must behave. For example, there exist parameters to state different connection options, such as the IP address of the message brokers, their listening port, and the topic to subscribe. For instance, for the Iris example introduced in Section 4.1, the *FLEX-consumer* produced by Flextory can be run following different execution modes. All the invocations have the same structure: “java -jar <*FLEX-consumer* name> -ip <broker address> -t <topic name> <optional arguments>”. The optional arguments offer very different customization options. For example, with “-mr 300 -d”, the *FLEX-consumer* of Iris will count the number of flowers of each species every 300 received messages, deleting the current messages after they have been processed, i.e., it will only count the new data that have arrived in the last 300 messages. Another possibility is “-tr 4 -w 8”, which indicates “count the number of each Iris species every 4 min and stop the *FLEX-consumer* execution if there are no new messages after 8 min since the last one received”. Note that, in this case, the messages will not be deleted after being processed, meaning that all the received messages will be processed each time.

**Figure 10:**
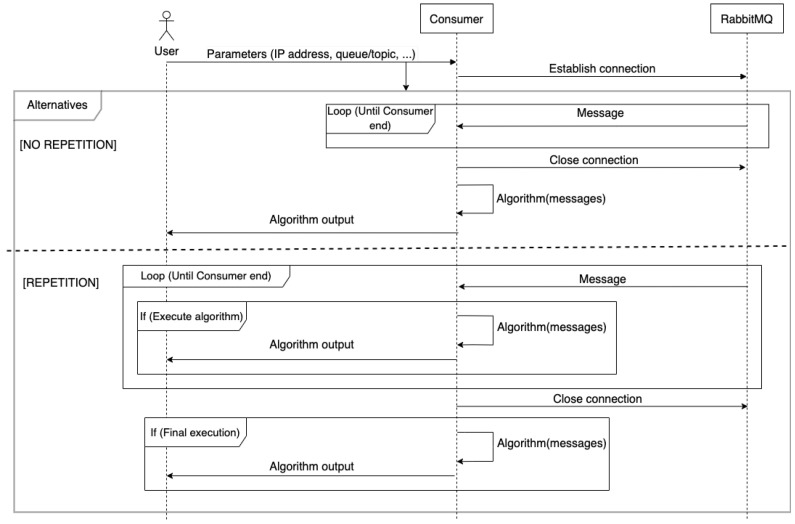
Message exchange between the *FLEX-consumer*, its user, and the message broker.

Finally, Figure 10 describes the lifecycle of a *FLEX-consumer*. In order to connect to the message broker (in the example RabbitMQ), the user provides the networking configuration (e.g., IP address and port of message broker, the topic name). In addition, the user can also define other configurable options such as the termination condition. Then, the *FLEX-consumer* establishes the connection with the broker and, depending on the configuration used, waits until a repetition or halt condition is triggered. *FLEX-consumers* can behave as long-lived connection applications, i.e., they can be configured without halting conditions, using the persistent option to maintain the execution of the processing algorithm indefinitely. It is worth mentioning that there are some constraints in the combination of some of these parameters. For instance, in the networking configuration, it is mandatory to have at least the IP address of the message broker and the topic (or queue in AMQP) to subscribe. In addition, it seems natural that the processing algorithm is executed at least once. Therefore, if no repetition parameters or the persistent option are specified, a halting condition must be specified. This way, the *FLEX-consumer* could execute the algorithm once and finish. Furthermore, if there is a repetition argument, there is no need to define a halting condition of the *FLEX-consumer*, although they can also be combined.

## 5. Illustrative Examples

In this section, we present two examples in which Flextory can help boost the development of a *FLEX-consumer*. Both examples arise from the needs of real research projects in which the authors currently participate. Flextory and all the material required to replicate these examples is published in a gitLab repository (https://gitlab.com/morse-uma/formal-methods/flextory/, accessed on 28 March 2024).

### 5.1. Learning from Observations

In the last few years, there has been rising interest in the so-called *digital twins*, that is, system models that can be enriched when new systems’ behaviors are observed. These models can be used to make decisions or predict failures. In order to construct a digital twin, a lot of information has to be collected and concurrently processed using a learning algorithm. The LearnFDT project aims to automatically generate *formal digital twins*, i.e., models of systems described with a formal language, using Automata Learning techniques. In this example, we use Flextory to generate a *FLEX-consumer* that constructs such formal digital twins.

In particular, the system to be learned is DASH [12], a protocol for adaptive video transmission. Thus, two entities are involved in DASH: a streaming video server and a client application. To learn the behavior of DASH, the *FLEX-consumer* application implements an algorithm based on Automata Learning techniques with passive learning [30]. The purpose of Automata Learning techniques is to build formal models that simulate the behavior of the systems under learning (SULs). The *passive* learning approach uses the *observed* behavior (execution traces) of the SUL to build the formal models.

**Figure 11:**
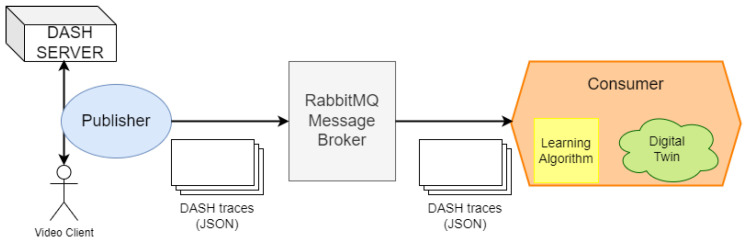
Deployment of the DASH case study with a DASH server acting as the publisher, a RabbitMQ message broker, and a *FLEX-consumer* instance integrating a learning algorithm.

Figure 11 shows a general overview of the case study. The objective is to generate a *FLEX-consumer* that is able to construct a digital twin of a DASH remote server. The setup for generating the digital twin consists of a publisher, a message broker, and the *FLEX-consumer*. The publisher sniffs traffic exchanged between the DASH server (available online [31]) and some clients during the execution of several video streaming sessions. Then, these traffic captures are packed in a message in JSON format and transmitted to the message broker. Since the publisher is beyond the scope of this work, we use a dummy publisher that reads the traces from a file and sends them to the broker. In this example, the message broker is a RabbitMQ instance that uses MQTT and has a topic “dash” where all DASH traces will be stored.

The *FLEX-consumer* receives network traces and executes the learning algorithm in order to incrementally produce a model of the DASH protocol. In this case study, we have the role of developers (the Flextory users) and also users of the *FLEX-consumer*. First, as developers, we provide Flextory with the JSON schema defining the traces format (see Appendix A for JSON schema definition). Then, we provide the *Algorithm* class that launches the learning algorithm and feeds it with incoming traces. The algorithm constructs a model of the system in an incremental manner, extending the learned model when new behaviors are read. The *Algorithm* class also deploys a web server that allows us to inspect the model under construction. We have integrated a *learning automata* algorithm, in which the system models are described as *timed automata* with one timer. The details of the learning algorithm are beyond the scope of this paper, but, in general, we can integrate any learning algorithm by injecting it as a dependency. Finally, we select MQTT as the communicating protocol in order to communicate with the RabbitMQ broker.

**Figure 12:**
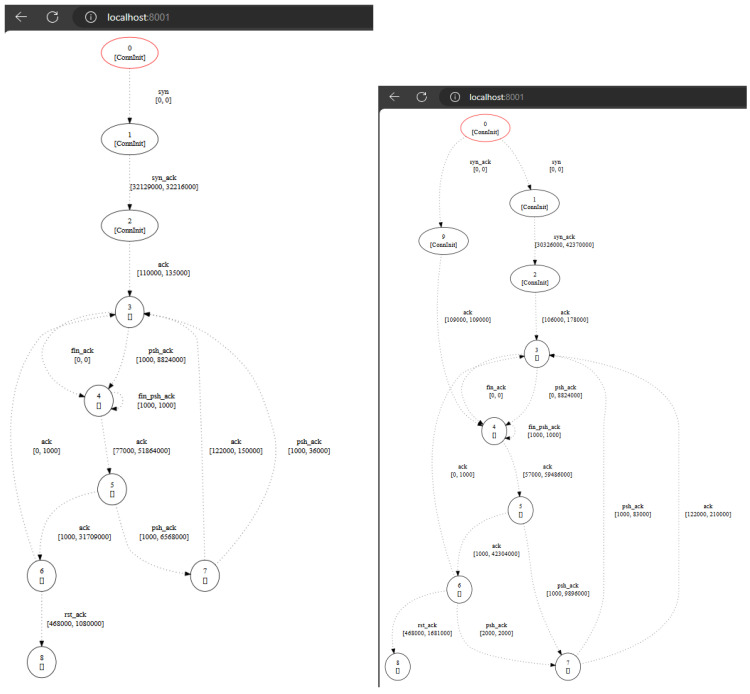
Intermediate automaton (**left**) and final automaton after learning 92 traces (**right**).

Once the *FLEX-consumer* is built, it is invoked with the following configuration: “java -jar Dash.jar -ip <ip address of the message broker> -pers -t dash”; that is, the *FLEX-consumer* is configured to establish a connection with the message broker, subscribe to the topic “dash”, and execute the processing algorithm in a persistent way. Then, the *FLEX-consumer* will start the connection and wait for new data. As mentioned before, the processing algorithm deploys a web server to check the progress of the Automata Learning algorithm. Figure 12 shows the timed automata learned during the *FLEX-consumer* execution (left) and the final automata produced after processing 92 traces.

### 5.2. Validating Data Format

The EPICENTRE project [32] proposes a 5G distributed experimentation platform. The platform, whose architecture is beyond the scope of this paper, includes a RabbitMQ broker with multiple queues. The first queue is used to inject the results of the experiments. These data are processed by a *consumer* application (called *Validator* in the project) that collects messages with a correct data format and injects them into a second queue in order to be processed by different analytics modules, which can also be considered as *consumer* applications. The broker communicates with all these entities using the MQTT protocol. In this project, most of these *consumers* (the *Validator* and the analytic modules) have been developed in Python. Anyhow, the programming language of the *consumer* is transparent to the broker message.

In this example, we use Flextory to generate a Java *Validator* so that it subscribes to the first queue and collects the correct messages. We have limited this example to the *Validator*, since it is the module developed by our research group. However, the rest of the other analytic modules used in the 5G-EPICENTRE project could also be generated using Flextory.

The development of the *Validator* follows the workflow of Flextory. In the first step, we upload the JSON schema and in the second step, we provide the *Algorithm* class. Both the definition of the JSON schema and the implementation of the *Algorithm* class are included in Appendix A. In this case, the *Validator* just logs in a file whether the messages are either correct or not. Since the *Algorithm* class does not include third-party libraries, we can directly move to the fourth step, in which we select the MQTT protocol. The last step is the compilation of the *FLEX-consumer* that finishes without reporting errors.

Finally, we have executed the generated *FLEX-consumer* to collect real data from the EPICENTRE platform. In particular, we configure the *FLEX-consumer* to check the format of each message when it is received and to stop (terminate execution) when 100 messages have been processed. The results have been quite satisfactory; the *Validator* created is similar to the original one, and the time required to produce it is minimal once the validation algorithm is coded in Java.

## 6. Evaluation: User Study

**Figure 13:**
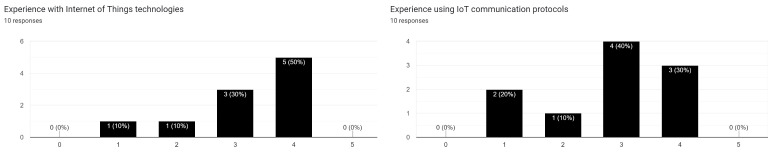
Participants’ experience in IoT technologies (**left**) and IoT communication protocols (**right**).

We have conducted a user study with ten participants recruited from post-graduate students with different knowledge levels in IoT technologies and communication protocols (see Figure 13). In the study, each participant is assigned an exercise that consists of (1) creating a *FLEX-consumer* using Flextory and (2) deploying and using it in a real environment. The *FLEX-consumer* has to process a sequence of messages coming from a message broker with information of different types of boats and use a machine learning algorithm to classify them. Finally, participants are requested to run the *FLEX-consumer* with different configurations. To reduce the time required to carry out the exercise, we have deployed a message broker that will interact with the resulting *FLEX-consumer*. Thus, the participants have to focus only on creating the consumer and using it. In order to successfully complete the implementation of the *FLEX-consumer*, they can follow the tutorial of Flextory (https://gitlab.com/morse-uma/formal-methods/flextory/, accessed on 28 March 2024). In case a participant is not familiar with some of the technology (e.g., specifying a JSON schema), we provide some backup material.

**Figure 14:**
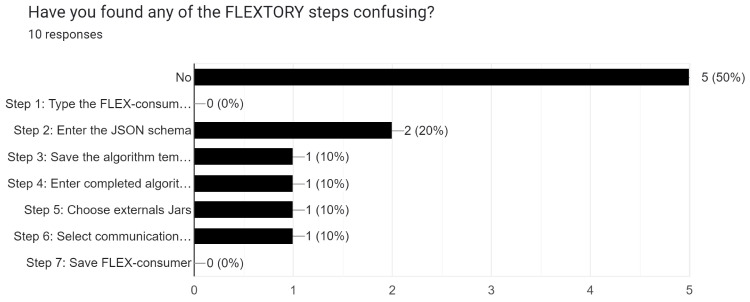
Users’ opinions on Flextory’s most complex steps.

We have collected the users’ opinions and suggestions using an online questionnaire (https://forms.gle/DwBEA7mUw1jkw5Zv9, accessed on 28 March 2024). In general, users with a higher knowledge in IoT give a very positive feedback of Flextory, whereas users with less experience do not have a clear picture of the utility of Flextory and *FLEX-consumers*. The conclusions of the user study are summarized as follows:

- All participants have been able to complete the practical task correctly. On average, the task was completed in less than 1 h. A total of 90% of participants think that Flextory is user-friendly and eases the task of implementing IoT consumers.
- As a possible improvement, some participants have suggested easing the installation process of Flextory by automatically installing the Java Development Kit (JDK) if it is not present on the machine. We believe that manually installing the JDK is not a big deal and allows more flexibility to decide which distribution to use.
- We explicitly asked about the most confusing step when using Flextory. As shown in Figure 14, there is not a consensus: 50% of the participants have not faced any issues using Flextory whereas 20% found some difficulties with the definition of the JSON schema.
- With respect to *FLEX-consumers*, 100% of users believe that they are easy to configure and use. However, 30% of them are not sure if they are useful. We believe that this may be related to the case study proposed in the exercise that could not be relevant or attractive enough. One participant suggested to generate *FLEX-consumers* to collect network data and perform a characterization of its behavior.
- Finally, we asked participants suggestions. Among others, they proposed to improve the documentation of Flextory and *FLEX-consumers*. In addition, they recommended to run Flextory as a web service in such a way that no installation is required. Finally, they suggested the integration of *FLEX-consumers* with other message brokers such as Kafka. Since Kafka uses its own communication protocol, we think that this proposal implies to design the templates for a new *FLEX-consumer* that implements the protocol.

## 7. Discussion

In this section, we discuss the pros and cons of Flextory and the *FLEX-consumers*. First, we would like to highlight that the target users of Flextory are developers without extensive background in IoT communication protocols who want to process data. In this case, Flextory is a valuable tool to produce, in *five steps,* a fully operational IoT consumer (*FLEX-consumer*) which is able to process data coming from a remote message broker. Since the IoT ecosystem is very diverse and changing, the *FLEX-consumer* execution mode can be re-configured without having to re-run Flextory. Thus, a consumer can be used in different scenarios. Clearly, this greatly simplifies the development cycle, and the results of the user study (see Section 6) indicate that, in general, potential users of Flextory are satisfied with it.

However, an expert user could find Flextory a bit limited. The main weakness of the generated *FLEX-consumers* is, presumably, that it can only receive data from one message broker and from one topic (or queue). For instance, in [33], the authors present an IoT Edge-Cloud hybrid architecture in which consumers have to dynamically connect to different message brokers according to different conditions. In order to adapt *FLEX-consumers* to new IoT architectures or even to a changing environment, we can adopt solutions from the SPL community, such as [5,6], that require different models (e.g., variability and goal models) to generate IoT applications. It should be noted that these models can be very dependent on the case study and therefore the Flextoryuser would need some knowledge of the case study and modeling techniques.

Another weak point could be the limited set of execution modes of the *FLEX-consumers*. Based on the literature, Flextory covers the most common execution modes, such as daemon mode and end processing after a time deadline. In addition, it is possible to set the frequency of execution of the processing algorithm according to the elapsed time or the number of messages received.

To finalize, the *FLEX-consumers* only support messages in JSON format. Although this format it is very flexible, thanks to the definition of the JSON schema, most message brokers support other formats. For example, RabbitMQ currently supports XML, Thrift, and MessagePack. Despite these limitations, Flextory could be useful for fast prototyping *FLEX-consumers*.

Concerning the IoT communication protocols integrated in *FLEX-consumers*, MQTT was a clear option for many reasons. In general, determining the most proper IoT communication protocol to use in a particular application is an important engineering problem, as many factors have to be considered. Since IoT devices have limited hardware features, a wide variety of IoT communication protocols have been developed to overcome distinct application problems while aiming for low latency, maximum throughput, and low energy consumption. Different studies [34,35] have compared the characteristics and capabilities of the most popular protocols, such as MQTT, HTTP, COAP, AMQP, and XMPP. There is usually a consensus about MQTT being the most suitable option for the majority of the IoT case studies. Even if it is not the option with the best performance, the strong points of the MQTT protocol are its lightweight design and the fact that a message can be sent to multiple subscribers with maximum performance, thanks the publisher/subscriber model. Moreover, MQTT is often preferred when a secure communication environment is needed. In the design phase of Flextory, our intention was to choose multiple communication protocols with features similar to MQTT’s. We selected AMQP because it can behave in a manner quite similar to MQTT, enabling the same features in both implementations. In further Flextory versions, we will study how to implement other protocols without changing the similar use of the *FLEX-consumers*.

## 8. Conclusions and Future Work

In this paper, we have presented Flextory, a software factory tool whose objective is to simplify the challenging and time-consuming task of implementing IoT data *consumer* applications. Although there exist some frameworks that ease the implementation process, they do not address the heterogeneous case studies of the IoT domain and, in many cases, developers are forced to build ad hoc applications. For this reason, we propose Flextory to guide a developer in the process of generating *consumer* applications that are characterized by connecting to a *message broker* and process received data. Thanks to Flextory developers do not have to worry about implementation details such as the communication logic with the message broker, the integration of the processing algorithm in the *FLEX-consumer*, or the management of the incoming data. In addition, Flextory produces configurable *FLEX-consumer* applications with a flexible behavior, in the sense that a *FLEX-consumer* user can define different conditions to trigger the processing algorithm or to finish the execution.

The current version of Flextory produces Java *FLEX-consumers* that support MQTT or AMQP communication protocols. This paper presents the requirements and architectures of both the software factory Flextory and the resulting *FLEX-consumers*.

To show the versatility of Flextory and the *FLEX-consumers* generated, we have presented a running example to describe the methodology (Section 4.1) and a more complex case study in Section 5. In addition to these examples, Flextory has also been used in other domains to show the wide variety of message formats and processing algorithms that can be included in the *FLEX-consumers*. In the field of computational phylogenetics, we have generated a *FLEX-consumer* that processes phylogenetic trees using the Sankoff [36,37] algorithm. Another case study is a *FLEX-consumer* that checks if data have been properly encoded using different cryptography algorithms. Finally, Flextory has been used to build a *FLEX-consumer* application that collects the result messages from different experiments and transforms them into a specific data format, which can be found at gitLab.

To assess the user’s opinion about Flextory, we have carried out a user study. The overall feedback is very positive, and the participants have made some suggestions that we plan to address.

As future work, we plan to extend Flextory in different ways to generate more flexible *FLEX-consumers* that can be used in different contexts. For instance, we have observed the importance of enabling subscription to multiple topics or queues. In addition, we would like to produce *FLEX-consumers* in Python, since it is a user-friendly language for non-software experts and has plenty of support to develop data analytics tools. Moreover, we would like to distribute Flextory as a web application or even as an Integrated Development Environment (IDE) plugin.

We also aim to check the compatibility of the current *FLEX-consumers* with other message brokers. Although AMQP and MQTT are standardized protocols, there are different versions, and some compatibility issues may occur depending on the version used in the *FLEX-consumer* application and the message broker.

In recent years, new IoT architectures have arisen from the evolution of wireless and mobile networks and require new features in the IoT applications. We plan to study in depth these new architectures in order to generate *FLEX-consumers* suitable for these dynamic scenarios.

## Figures and Tables

**Table 1:** Features of different Message brokers.

Name	Business Model	Hosting	Native Protocols	Additional Information
RabbitMQ [18]	Open Source	Self-hosting	AMQP	Plugin support including MQTT extension
Azure IoT Hub [19]	Proprietary	Cloud hosting	HTTP, AMQP, and MQTT	Integration with Azure services
Apache Kafka [20]	Open Source	Self-hosting	TCP-based protocol	Supports AMQP and MQTT via plugin
Eclipse Mosquitto [21]	Open Source	Self-hosting	MQTT	-
AWS IoT Core [22]	Proprietary	Cloud hosting	MQTT and HTTPS	Integration with AWS and other Amazon services

**Table 2:** Functional and non-functional requirements of Flextory.

Id	Description
FR-1	Developers have to specify the format of the data received by the *FLEX-consumer*.
FR-2	Flextory will have a way to enter the algorithm that the *FLEX-consumer* will execute to process the received data.
FR-3	Flextory should offer a template of the processing algorithm that users have to complete.
FR-4	Flextory will allow the user to add external dependencies of the *FLEX-consumer*’s processing algorithm in JAR format.
FR-5	Developers have to select either MQTT or AMQP as the *consumer*’s protocol, but not both.
FR-6	The name of the resulting *FLEX-consumer* has to be configured by the user.
FR-7	Flextory has to give warning messages to users when an error occurs.
NFR-1	Flextory must have an intuitive and easy-to-use GUI.
NFR-2	Flextory should be offered as a stand-alone application in JAR format.
NFR-3	Flextory should be compatible with several operating systems.
NFR-4	Flextory must be executable on systems that have the Java Development Kit (Java JDK).

**Table 3:** Functional and non-functional requirements of *FLEX-consumers*.

Id	Description
FR-1	A *FLEX-consumer* has to connect to a message broker using AMQP or MQTT protocols.
FR-2	The IP address and port of the message broker has to be configurable.
FR-3	*FLEX-consumers* should be able to subscribe to a specific queue or topic depending on the protocol.
FR-4	The topic or the queue name to be subscribed has to be configurable.
FR-5	*FLEX-consumers* must include configurable trigger conditions to control the processing algorithm execution.
FR-6	A *FLEX-consumer* user can configure whether messages are discarded once processed or they continue to be processed in subsequent calls to the algorithm.
FR-7	There should be an option to decide if the processing algorithm can be executed one last time before closing the connection with the message broker.
FR-8	*FLEX-consumers* will offer options to configure when their execution stops.
FR-9	*FLEX-consumers* must include an option to invoke the processing algorithm at the beginning of the lifecycle.
FR-10	*FLEX-consumers* should include an error reporting system.
FR-11	*FLEX-consumers* should provide help or usage information to users.
NFR-1	*FLEX-consumers* must be executable on a system that has the Java Development Kit (Java JDK).
NFR-2	The *FLEX-consumer*’s user interface should be user friendly.

**Figure A1:**
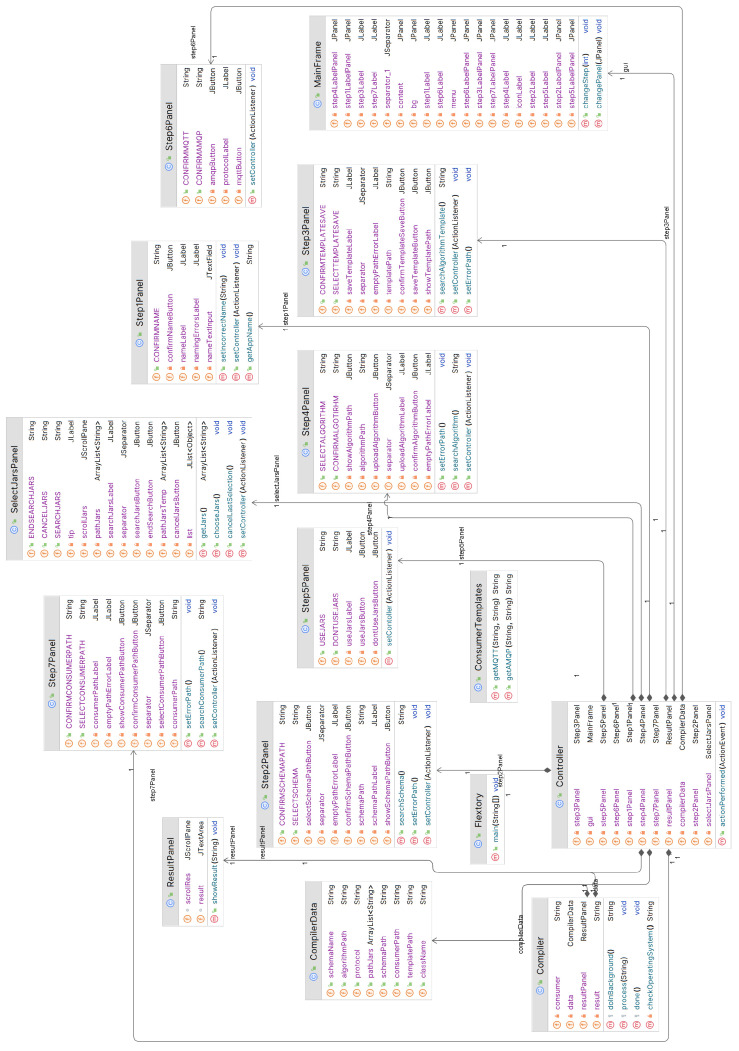
Flextory class diagram.

## Data Availability

Data is contained within the article.

## Appendix A

This appendix includes supplementary material to clarify the implementation of Flextory and the examples presented in Section 4.1 and Section 5.

Figure A1 shows the class diagram of the current implementation of Flextory, which is related to the components diagram shown in Figure 6. Classes with suffixes Panel and Frame are part of the View component. The ConsumerTemplates and Compiler classes are part of the Model component. The former class includes the code of a generic MQTT and AMQP client in String format. The AMQP client’s template code uses an external library provided by RabbitMQ [38], while the MQTT client relies on the Eclipse Paho library [39]. The Compiler class includes methods to generate and compile all the Java code of the *FLEX-consumer* and package the result in JAR format. For instance, to convert the JSON schema into serializable classes, Flextory uses the Jackson library (https://github.com/FasterXML/jackson, accessed on 28 March 2024). Finally, the Controller class includes all the events handlers in order to properly update the View and the Model.

Listings A1 and A2 show part of the code of the AMQP and MQTT *FLEX-consumers*. We would like to clarify that the main differences between them only concern the communication module. As mentioned above, the AMQP template (Listing A1) uses the RabbitMQ library that supports AMQP communications. In Figure A1, the method connect (lines 22–53) is in charge of establishing the connection with the broker to create a channel to receive the messages of a specific queue. In addition, we have to define a callback (deliverCallback lines 34–51) that will be executed when a new message arrives. Basically, depending on the configuration, this callback will launch the processing algorithm in a worker thread or will delete the queue, close the connection with the broker, and end the *FLEX-consumer* execution. The method end (lines 55–64) implements this functionality.

**Listing A1.** Communication module of an AMQP *FLEX-consumer*.

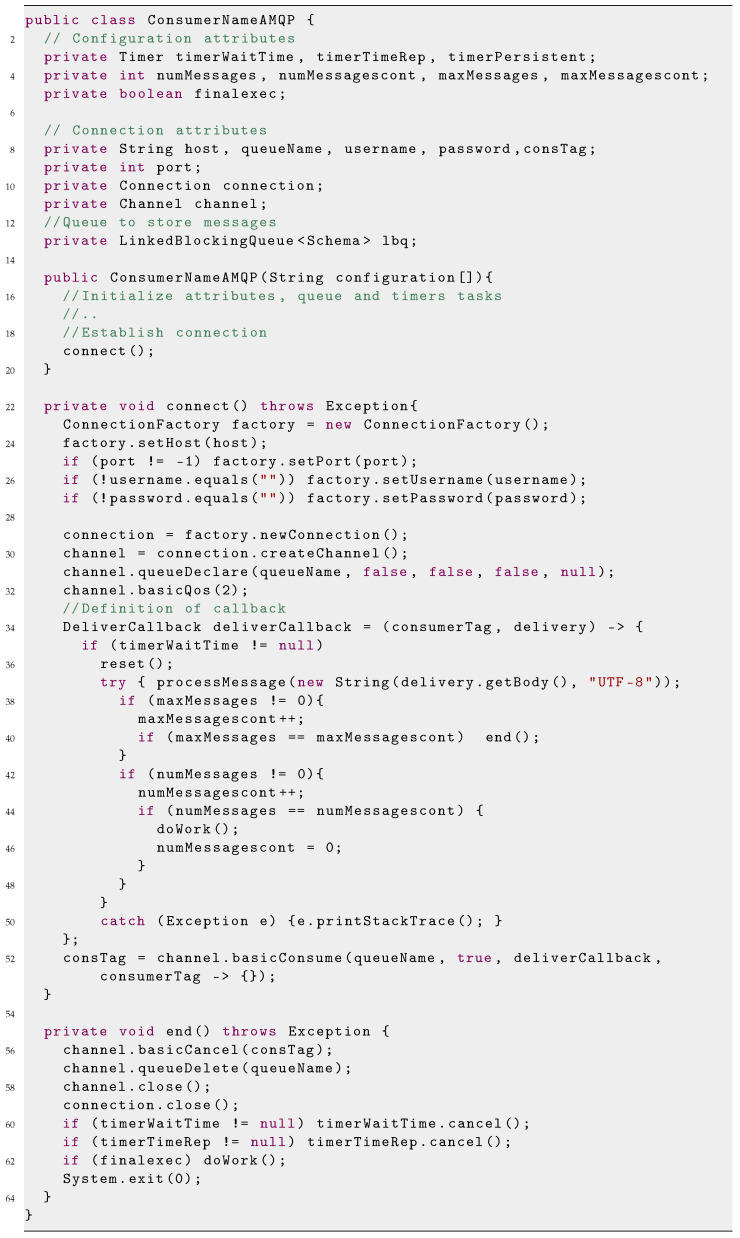

In the MQTT client shown in Figure A1, we use the Eclipse Paho library. In this case, the connect method (lines 16–27) creates a session with the broker and subscribes to a specific topic. When a new message arrives, it is managed by the messageArrived method (lines 29–54). Observe that its implementation is similar to deliveryCallback of the AMQP template. Finally, the method end (lines 56–63) closes the session with the broker and ends the execution of the client.

**Listing A2.** Communication module of an MQTT *FLEX-consumer*.

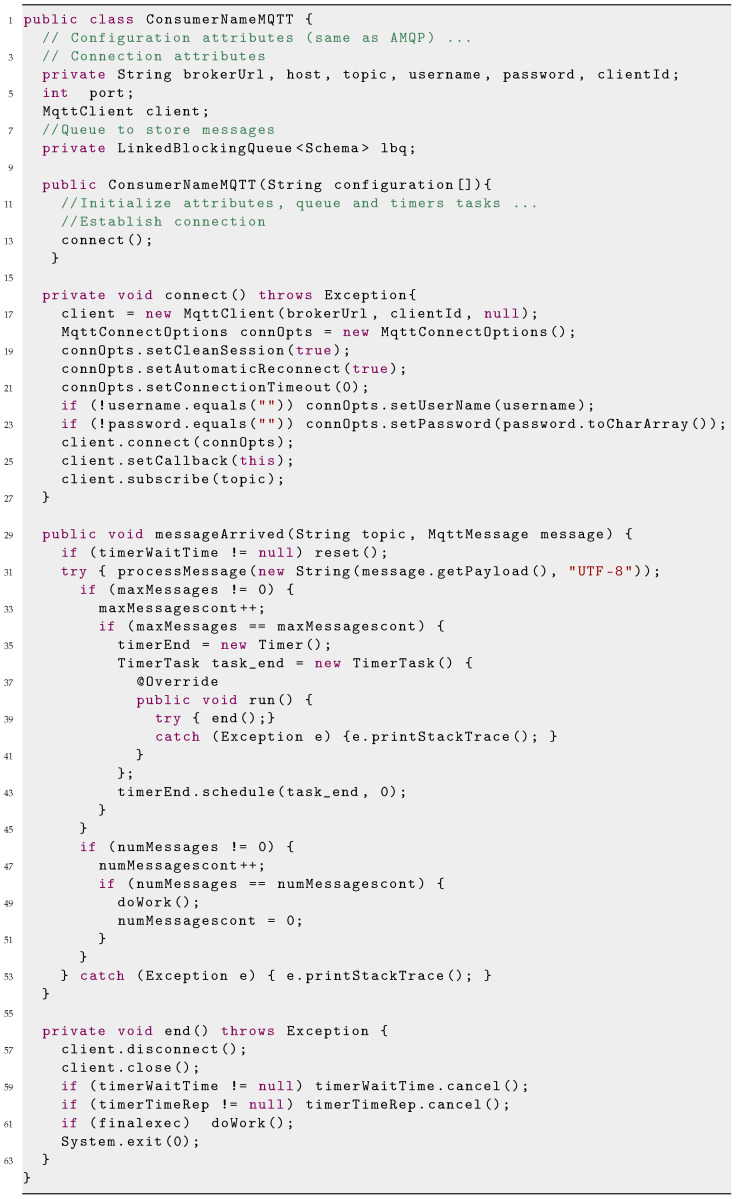

We continue with the definition of the JSON schema of the examples presented in Section 4.1 and Section 5. JavaScript Object Notation (JSON) is a lightweight data-interchange format commonly used for communication in the IoT domain. A JSON schema (https://json-schema.org/, accessed on 28 March 2024) provides a formal description of the expected format of JSON messages, including the data type of each field, any constraints on the data, and the relationships between different parts of the message. Using a JSON schema allows for validation of incoming and outgoing messages.

Listing A3 shows the JSON schemas used in the DASH example (left) and the Validator example (right). Both schemas include the fields title, type, properties, and additionalProperties. However, in each example, the messages include a different set of properties. For example, the DASH example includes information of the TCP header, such as the flags activated (flags) or the sequence number (seqN). In the Validator example, the properties include information of the experiments such as the experiment id or the scenario, among others.

**Listing A3.** JSON schemas of the DASH example (**left**) and the Validator example (**right**).

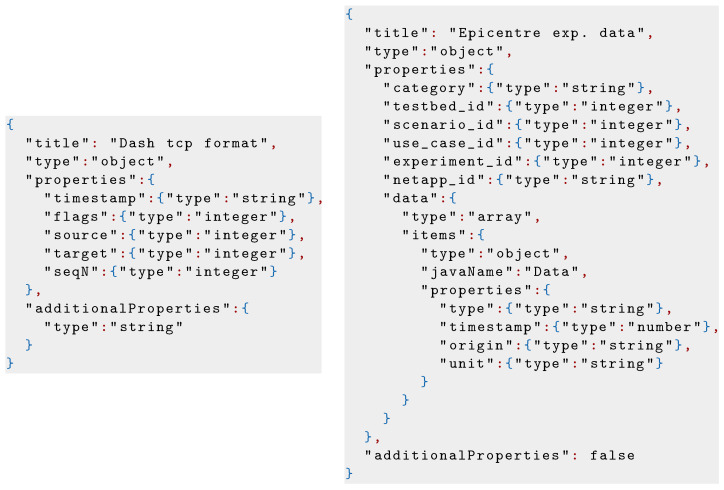

To conclude, we present the implementation of the *Algorithm* class used in the Validator case study (Listing A4). The *Algorithm* class implements the Runnable interface so that the processing algorithm can be executed in a different thread. The processing algorithm is coded in the run method and can include references to external libraries. In the example, it only logs in a file that the messages are either correct or not. Observe that the run method processes messages from the class attribute data (line 14), which consist of a queue where the communication module stores all incoming messages.

**Listing A4.** Implementation of the Algorithm class of the Validator consumer.

## References

[1] Hassan R., Qamar F., Hasan M.K., Aman A.H.M., Ahmed A.S. (2020). Internet of Things and Its Applications: A Comprehensive Survey. Symmetry.

[2] Advanced Message Queuing Protocol. https://www.amqp.org/.

[3] MQTT: The Standard for IoT Messaging. https://mqtt.org/mqtt-specification/.

[4] Pohl K., Böckle G., Van Der Linden F. (2005). Software Product Line Engineering: Foundations, Principles, and Techniques.

[5] Butting A., Kirchhof J.C., Kleiss A., Michael J., Orlov R., Rumpe B. (2022). Model-Driven IoT App Stores: Deploying Customizable Software Products to Heterogeneous Devices. Proceedings of the 21st ACM SIGPLAN International Conference on Generative Programming: Concepts and Experiences (GPCE 2022).

[6] Ayala I., Amor M., Horcas J.M., Fuentes L. (2019). A goal-driven software product line approach for evolving multi-agent systems in the Internet of Things. Knowl. Based Syst..

[7] Cusumano M.A. (1989). The Software Factory: A Historical Interpretation. IEEE Softw..

[8] Greenfield J., Short K. (2003). Software factories: Assembling applications with patterns, models, frameworks and tools. Proceedings of the Companion of the 18th Annual ACM SIGPLAN Conference on Object-Oriented Programming, Systems, Languages, and Applications (OOPSLA’03).

[9] Benaddi L., Ouaddi C., Jakimi A., Ouchao B. (2024). Towards A Software Factory for Developing the Chatbots in Smart Tourism Mobile Applications. Procedia Comput. Sci..

[10] Beneke T. (2014). A Perfect Match: Java and the Internet of Things. https://www.oracle.com/technical-resources/articles/java/java-maker-iot.html.

[11] Angluin D. (1987). Learning regular sets from queries and counterexamples. Inf. Comput..

[12] International Organization for Standardization (ISO), I.E.C.I Dynamic Adaptive Streaming over HTTP (DASH). https://standards.iso.org/ittf/PubliclyAvailableStandards/index.html.

[13] MQTT Version 3.1.1. http://docs.oasis-open.org/mqtt/mqtt/v3.1.1/mqtt-v3.1.1.html.

[14] MQTT Version 5.0. http://docs.oasis-open.org/mqtt/mqtt/v5.0/os/mqtt-v5.0-os.html.

[15] OASIS Advanced Message Queuing Protocol (AMQP). https://docs.oasis-open.org/amqp/core/v1.0/os/amqp-core-overview-v1.0-os.html.

[16] The Constrained Application Protocol (CoAP). https://datatracker.ietf.org/doc/html/rfc7252.

[17] Extensible Messaging and Presence Protocol (XMPP): Address Format. https://datatracker.ietf.org/doc/rfc7622/.

[18] RabbitMQ. https://www.rabbitmq.com/.

[19] Azure IoT Hub. https://azure.microsoft.com/products/iot-hub.

[20] Apache Kafka. https://kafka.apache.org/.

[21] Eclipse Mosquitto. https://mosquitto.org/.

[22] AWS IoT Core. https://aws.amazon.com/iot-core/.

[23] de Souza Cimino L., de Resende J.E.E., Silva L.H.M., Rocha S.Q.S., de Oliveira Correia M., Monteiro G.S., de Souza Fernandes G.N., da Silva Moreira R., de Silva J.G., Santos M.I.B. (2019). A middleware solution for integrating and exploring IoT and HPC capabilities. Softw. Pract. Exp..

[24] Cherrier S., Ghamri-Doudane Y.M., Lohier S., Roussel G. D-LITe: Distributed logic for internet of things sErvices. Proceedings of the 2011 IEEE International Conferences on Internet of Things and Cyber, Physical and Social Computing, iThings/CPSCom 2011.

[25] Chauhan S., Patel P., Sureka A., Delicato F.C., Chaudhary S. Demonstration Abstract: IoTSuite—A Framework to Design, Implement, and Deploy IoT Applications. Proceedings of the 2016 15th ACM/IEEE International Conference on Information Processing in Sensor Networks (IPSN).

[26] Akasiadis C., Pitsilis V., Spyropoulos C.D. (2019). A Multi-Protocol IoT Platform Based on Open-Source Frameworks. Sensors.

[27] Nguyen X.T., Tran H.T., Baraki H., Geihs K. FRASAD: A framework for model-driven IoT Application Development. Proceedings of the IEEE World Forum on Internet of Things, WF-IoT 2015.

[28] Henelius A., Torniainen J. (2018). MIDAS: Open-source framework for distributed online analysis of data streams. SoftwareX.

[29] Iris Data Set. https://archive.ics.uci.edu/ml/datasets/iris.

[30] Aichernig B.K., Muškardin E., Pferscher A. (2022). Active vs. Passive: A Comparison of Automata Learning Paradigms for Network Protocols. Electron. Proc. Theor. Comput. Sci..

[31] DASH, HLS or PROGRESSIVE Stream Test. https://bitmovin.com/demos/stream-test?format=dash&manifest=https%3A%2F%2Fcdn.bitmovin.com%2Fcontent%2Fassets%2Fart-of-motion-dash-hls-progressive%2Fmpds%2Ff08e80da-bf1d-4e3d-8899-f0f6155f6efa.mpd.

[32] Arampatzis D., Apostolakis K.C., Margetis G., Stephanidis C., Atxutegi E., Amor M., Di Pietro N., Henriques J., Cordeiro L., Carapinha J. Unification architecture of cross-site 5G testbed resources for PPDR verticals. Proceedings of the 2021 IEEE International Mediterranean Conference on Communications and Networking, MeditCom 2021.

[33] Pham V.N., Lee G.W., Nguyen V., Huh E.N. (2021). Efficient Solution for Large-Scale IoT Applications with Proactive Edge-Cloud Publish/Subscribe Brokers Clustering. Sensors.

[34] Bayılmış C., Ebleme M.A., Çavuşoğlu Ü., Küçük K., Sevin A. (2022). A survey on communication protocols and performance evaluations for Internet of Things. Digit. Commun. Netw..

[35] Wytrębowicz J., Cabaj K., Krawiec J. (2021). Messaging Protocols for IoT Systems—A Pragmatic Comparison. Sensors.

[36] Sankoff D. (1975). Minimal Mutation Trees of Sequences. SIAM J. Appl. Math..

[37] Sankoff D., Rousseau P. (1975). Locating the vertices of a steiner tree in an arbitrary metric space. Math. Program..

[38] RabbitMQ Java Client Library. https://www.rabbitmq.com/java-client.html.

[39] Eclipse Paho Java Client. https://github.com/eclipse/paho.mqtt.java.

